# SNP-based and haplotype-based genome-wide association on drug dependence in Han Chinese

**DOI:** 10.1186/s12864-024-10117-4

**Published:** 2024-03-06

**Authors:** Hanli Xu, Yulin Kang, Tingming Liang, Sifen Lu, Xiaolin Xia, Zuhong Lu, Lingming Hu, Li Guo, Lishu Zhang, Jiaqiang Huang, Lin Ye, Peiye Jiang, Yi Liu, Li Xinyi, Jin Zhai, Zi Wang, Yangyang Liu

**Affiliations:** 1 College of Life Sciences and Bioengineering, School of Science, Beijing Jiaotong University, Beijing, 100028 China; 2 Chinese Research Academy of Environmental Sciences, Beijing, 100012 China; 3 Jiangsu Key Laboratory for Molecular and Medical Biotechnology, School of Life Science, Nanjing Normal University, Nanjing, 210023 China; 4 Precision Medicine Key Laboratory of Sichuan Province and Precision Medicine Center, West China Hospital, Sichuan University, Chengdu, 610041 China; 5 Office of Academic Affairs, The National Police University for Criminal Justice, Baoding, 071000 China; 6 School of Biological Science & Medical Engineering, Southeast University, Nanjing, 211189 China; 7 School of Geographic and Biologic Information, Nanjing University of Posts and Telecommunications, Nanjing, 210003 China; 8 Cheung Hong School of Journalism and Communication, Shantou University, Shantou, 515060 China; 9 Office of International Cooperation and Exchanges, Nanjing University, Nanjing, 210023 China; 10 Jiangsu Taihu Institute of Addiction Rehabilitation, Suzhou, 215111 China; 11 Department of Social Work, Changzhou University, Changzhou, 213164 China; 12 School of Music, Nanjing Normal University, Nanjing, 210097 China; 13 Department of Psychology, Nanjing University, Nanjing, 210023 China; 14 School of Education, Tianjin University, Tianjin, 200350 China

**Keywords:** Drug addiction, Genome-wide association, Single nucleotide polymorphism, Haplotype, Han Chinese

## Abstract

**Background:**

Drug addiction is a serious problem worldwide and is influenced by genetic factors. The present study aimed to investigate the association between genetics and drug addiction among Han Chinese.

**Methods:**

A total of 1000 Chinese users of illicit drugs and 9693 healthy controls were enrolled and underwent single nucleotide polymorphism (SNP)-based and haplotype-based association analyses via whole-genome genotyping.

**Results:**

Both single-SNP and haplotype tests revealed associations between illicit drug use and several immune-related genes in the major histocompatibility complex (MHC) region (SNP association: log_10_BF = 15.135, *p* = 1.054e-18; haplotype association: log_10_BF = 20.925, *p* = 2.065e-24). These genes may affect the risk of drug addiction via modulation of the neuroimmune system. The single-SNP test exclusively reported genome-wide significant associations between rs3782886 (SNP association: log_10_BF = 8.726, *p* = 4.842e-11) in *BRAP* and rs671 (SNP association: log_10_BF = 7.406, *p* = 9.333e-10) in *ALDH2* and drug addiction. The haplotype test exclusively reported a genome-wide significant association (haplotype association: log_10_BF = 7.607, *p* = 3.342e-11) between a region with allelic heterogeneity on chromosome 22 and drug addiction, which may be involved in the pathway of vitamin B12 transport and metabolism, indicating a causal link between lower vitamin B12 levels and methamphetamine addiction.

**Conclusions:**

These findings provide new insights into risk-modeling and the prevention and treatment of methamphetamine and heroin dependence, which may further contribute to potential novel therapeutic approaches.

**Supplementary Information:**

The online version contains supplementary material available at 10.1186/s12864-024-10117-4.

## What is already known on this topic

Drug addiction may be associated with genetic factors, and individuals with specific SNPs or haplotypes may have different levels of drug dependence following similar exposures.

### What this study adds

Both single-SNP and haplotype tests revealed associations between illicit drug use and several immune-related genes in the major histocompatibility complex (MHC) region, which may influence the risk of drug addiction through modulation of the neuroimmune system. Single-SNP analysis exclusively identified rs3782886 in *BRAP* and rs671 in *ALDH2* as showing significant genome-wide associations with drug addiction, while haplotype analysis exclusively reported a significant genome-wide association between a region with allelic heterogeneity on chromosome 22 and drug addiction, which may be involved in the pathway of vitamin B12 transport and metabolism.

### How this study might affect research, practice or policy

This study could provide new insights for risk-modeling and the prevention and treatment of methamphetamine and heroin dependence, which may further contribute to potential novel therapeutics.

## Introduction

Drug abuse and drug dependence, characterized by the consumption of illicit drugs or narcotics and inappropriate use of prescription drugs, imparts heavy burdens on the safety and well-being of individuals and on human society [1]. The number of drug addicts has been increasing every year, and the United Nations Office on Drugs and Crime (UNODC) reported that drug usage increased 30% globally from 2009 to 2018, with 35 million people fulfilling the criteria for a drug-use disorder in 2019 [2]. Over time, drug users encounter drug tolerance, cravings, and relapses caused by psychoneurological changes [3], potentially leading to serious physical, psychological, and social consequences, deteriorating quality of life, and impaired physical and psychological health [4]. Understanding the mechanisms underlying addiction and drug dependence will help scientists, physicians, and stakeholders to develop better public health preventive strategies and clinical treatments [5]; however, the detailed mechanisms that underlying the risks of drug dependence currently remain unclear.

The etiology of drug addiction involves impulsivity, anxiety, depression, and stress responsivity, as well as underlying genetic risks. Although environmental factors and curiosity are considered to be the most important reasons for initial drug use, genetic factors are thought to have a marked contribution to the addiction component [6, 7]. Based on the potential contributions of genetic variation, some GWAS have identified novel susceptibility genes and provided new insights into the etiology of MA and heroin dependence [8, 9]. Aldehyde dehydrogenase 2 (*ALDH2*) is a key enzyme in the oxidation process of acetaldehyde to acetate, which has been associated with an increased risk of drug addiction [10]. The *OPRM1* gene is also associated with drug addiction [11], and *OPRM1* rs1799971 was shown to be a genetic risk factor for drug addiction among Jordanian males. rs2133896 in *ANKS1B* is associated with its gene expression and with reduced the gray matter in the left calcarine and white matter in the right superior longitudinal fasciculus in individuals with heroin dependence [12]. *OPRD1* genetic variants were associated with methadone dose in individuals receiving methadone maintenance treatment for heroin dependence [13], and the *OPRM1* A118G polymorphism was found to be associated with opioid addiction in a Pakistani population. Although GWAS analyses have identified statistically reliable and robust genetic loci that are associated with drug dependence, only a small fraction of the phenotypic variation can be explained by this aberrations [14], known as “missing heritability” [15]. Current GWAS strategies usually involve the detection of common single nucleotide polymorphisms (SNPs), one SNP at a time; however, more phenotypic variation can be explained by analyzing SNPs jointly [16, 17]. Some relevant studies have identified potential SNPs associated with drug dependence, but fewer studies have revealed the potential addiction mechanisms for drug dependence, especially in the Chinese Han population. Moreover, haplotype-based GWAS may explain additional heritability and reveal previously unknown neurobiological pathways for drug dependence. Notably however, fewer studies have focused on SNP profiles and the haplotype distribution associated with drug addiction, which may be crucial for revealing the detailed molecular mechanisms and genetic susceptibility underlying drug abuse. In addition, current relevant research has been limited by small sample sizes, thus compromising their statistical power, and by a lack of studies in Han Chinese individuals, which includes a large addict population.

In this study, we aimed to analyze the SNP profile and haplotype distribution associated with drug addiction, and to explore the genetic etiology of MA and heroin use in the Chinese Han population, using a two-layer hidden Markov model for linkage disequilibrium (LD) and a Bayesian regression model. We also screened *BRAP* rs3782886 and *ALDH2* rs671, immune-related genes in the MHC and an LD block on chromosome 22 associated with drug dependence, which may help to reveal crucial susceptibility genes and thus provide novel insights for the prevention and treatment of MA and heroin dependence.

## Materials and methods

### Study participants

**Fig. 1:**
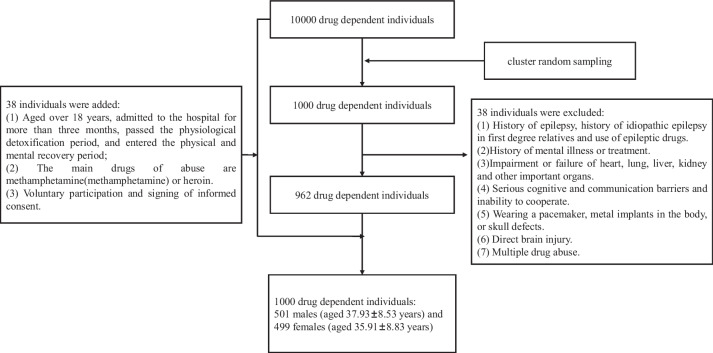
Flowchart of subject enrollment and follow-up

The flowchart for subject enrollment and follow-up is presented in Fig. 1. A total of 1000 drug-dependent individuals were recruited, including 501 males (aged 37.93 ± 8.53 years) and 499 females (aged 35.91 ± 8.83 years), who had been diagnosed with MA dependence or heroin dependence by professional staff in rehabilitation at Jiangsu Provincial Bureau of Drug Rehabilitation. Participants had no history of epilepsy and had not been diagnosed with any other major psychiatric illnesses, such as recurrent depression or schizophrenia. Among these, 919 (91.90%) used MA and 78 (7.80%) used heroin. We also included data from 9693 healthy controls in Jiangsu Province, China. The inclusion criteria were: (1) aged > 18 years, admitted to hospital for > 3 months, passed the physiological detoxification period, and entered the physical and mental recovery period; (2) main drugs of abuse MA or heroin; and (3) voluntary participation and signed informed consent. The exclusion criteria were: (1) history of epilepsy, history of idiopathic epilepsy in first-degree relative, or use of anti-epileptic drugs; (2) history of mental illness or treatment; (3) impairment or failure of heart, lung, liver, kidney, or other important organs; (4) serious cognitive or communication barriers and inability to cooperate; (5) having a pacemaker, metal implants in the body, or skull defects; (6) direct brain injury; and (7) multiple drug abuse. To minimize false-positive results related to population stratification, enrollment was limited to individuals of self-reported Han Chinese ancestry. The study was approved by Tianjin University institutional review board (No. TJUE-2021-014). All experiments were performed following relevant guidelines and regulations. Written informed consent was obtained from all participants. Peripheral blood samples were drawn from all participants at enrollment.

### Whole-genome genotyping and quality controls in the discovery stage

Genomic DNA was extracted from whole-blood samples from each subject using a commercial DNA extraction kit. Quantitation and quality control of the extracted DNA were performed using a spectrophotometer using Pico-green dsDNA Reagent. Genome-wide genotyping was performed using an Infinium Asian Screening Array (Illumina, Inc., San Diego, CA, USA) following the manufacturer’s instructions. This array included 696,080 SNP markers, and was specifically designed for East Asian individuals. Genotype call rate was > 0.9 in each subject.

Pairwise identity by descent estimation was performed using PLINK 1.9 [18], which was used to detect pairs of individuals who were more closely related than would be expected in a random study. One individual was excluded from each closely related pair with PI_HAT > 0.125, and the remaining 9145 individuals (962 cases and 8183 controls) were deemed to represent independent cases. Based on these individuals, we further excluded SNPs if the Hardy-Weinberg equilibrium exact test *p*-value was < 1.00e-6, if the minor allele frequency was < 1%, or if the proportion of missing genotypes was > 5%. The final number of autosome SNPs used for the association analysis was 467,292.

### Controlling of population stratification

SNPs were selected for principal component analysis (PCA) by thinning the full set of autosomal SNPs so that the remaining SNPs spaced were at least 0.001 centimorgans apart (estimated by HapMap). After that, SNPs located in the MHC, the lactase region (2q21), and known inversions of 8p23 and 17q21.31 were also removed. A total of 238,230 SNPs were therefore analyzed by PCA. No obvious outlier individuals were detected, and the top 10 principal components (eigenvectors) were used to control population stratification in the regression model to estimate associations.

### Association analysis

Association tests of single SNPs and local haplotypes were performed using hapQTL [18], using the following regression model (1):

1 $$Y= W\alpha + L\beta +e$$

where, y = (y_1_, …, y_n_) is a vector of phenotypic values of drug dependence, W is an n × q matrix representing q covariates (such as sex and principal components for population stratification), e ~ MVN(0, τ^−1^I_n_) is the error term, I_m_ denotes an identity matrix of dimension m, and MVN is the multivariate normal distribution. L is a vector or matrix of genotype, which may be a single SNP or haplotype. A Bayesian approach was used to fit the regression model. Bayes factors were used for statistical analysis because Bayes factors of different genetic loci can be compared and ranked, while *p* values for different loci had different null hypotheses and could not be compared.

### Genetic correlations with other psychiatric phenotypes

We used publicly available recent GWAS results from the Psychiatric Genomics Consortium on major depressive disorder (MDD), bipolar disorder (BIP), cannabis use disorder, and alcohol dependence, and carried out LD score regression using the R package Genomic SEM [19] to estimate the genetic correlation between our results and the above GWAS results.

### Functional analysis of screened genes

To understand the potential functional implications of the involved genes with candidate SNPs, functional enrichment analysis was exemplified with The Database for Annotation, Visualization, and Integrated Discovery (DAVID) version 6.8 [20], We also performed relevant functional analysis using Metascape [21] and queried for potential contributions in Kyoto Encyclopedia of Genes and Genomes (KEGG) pathways.

### Experimental validation of SNPs

We performed experimental validation of the screened candidate SNPs associated with drug addiction (rs3782886 in *BRAP* and rs671 in *ALDH2*) using oral samples from 80 cases, including 40 male and 40 female participants with drug dependence. Total DNA was extracted using a Genome Extraction Kit (Tiangen, Beijing, China), and the primers were designed based on the SNPs of interest. The extracted DNA concentrations were evaluated using an Agilent 2200 TapeStation system. The primer sequences were: *BRAP* forward 5′- ACACGTCCATCACTTCCA-3′ and reverse 5′-GATTAGCCTACCAATCAG-3′, and *ALDH2* forward 5′-GACCATAGAGGAGGTTGTT-3′ and reverse 5′-CTCTTACCCTCAGCCAAC-3′. The DNA was then amplified by polymerase chain reaction (PCR) in a 50 μL volume, including 2 μL Primer F (10 μM), 2 μL Primer R (10 μM), 25 μL 2× Rapid Taq Master Mix, 2 ng template DNA, and ddH2O up to 50 μL. The PCR cycling parameters were as follows:

**Table:** 

95 °C	3 min	
35 cycles:		
	95 °C	15 sec
	54 °C for *BRAP* and 56 °C for *ALDH2*	15 sec/kb
	72 °C	15 sec
72 °C	5 min	

The primer lengths for *BRAP* and *ALDH2* are 18 bp and 19 bp, respectively

Following PCR amplification, the purified product was sequenced by Shanghai Jieli Biological Technology Co., Ltd.

### Statistical analysis

Unpaired t-tests and Wilcoxon’s rank-sum tests were used for hypothesis testing of unpaired numeric samples and potential differences among groups were estimated by one-way analysis of variance (ANOVA). Other relevant statistical analyses are described under the relevant methods. All statistical analyses were carried out using R (version 4.0.5), and Venn distributions were imaged using a publicly available tool (http://bioinformatics.psb.ugent.be/webtools/Venn/).

## Results

### Genome-wide analysis

All detected SNPs were first analyzed using PLINK, only the significant SNPs (*p* ≤ 9.96e-07) were screened. A total of 245 (74.47%) SNPs were found on chromosome 6 (mainly MHC region), with only 84 (25.53%) were located on other chromosomes, intergenic SNPs were the most dominant, followed by intronic, ncRNA intronic, and exonic SNPs. On chromosome 6, intergenic SNPs were the most dominant SNPs, followed by ncRNA intronic SNPs, and intronic and exonic SNPs (Fig. S1A).

### Identification of loci with significant associations with drug addiction

**Fig. 2:**
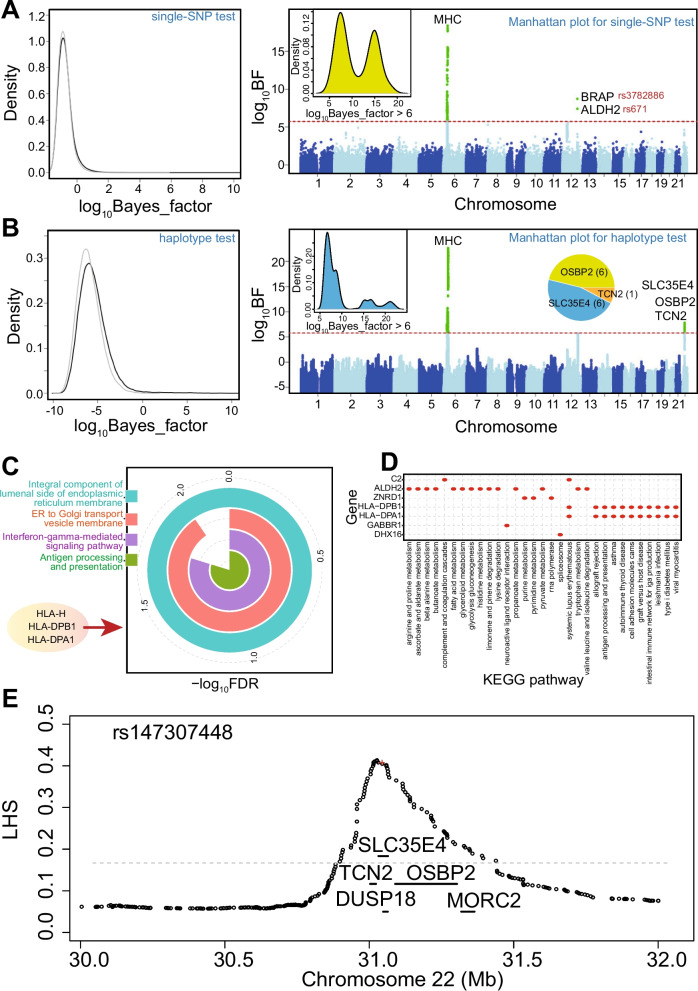
Distributions of single-SNP and haplotype tests. **A**, **B** Null (gray line) and alternative (black line) distributions of Bayes factors for single SNP and haplotype and their Manhattan plots. Log_10_BF values under alternative distributions truncated at 10; null distribution obtained by permuting the phenotype once. Alternative distributions obtained using true phenotype. **C** Functional analysis of involved genes in (A) with > 6 log_10_BF (*n* = 17). Upper image shows significantly enriched GO terms (FDR < 0.05); biological processes mainly involved three genes: *HLA-H*, *HLA-DPB1*, and *HLA-DPA1*. Lower image shows distribution of involved KEGG pathways for involved genes. **D** Examples of genome-wide significant haplotype associations. LD block (based on local haplotype sharing) around each significant marker of same region (red “+”) denoted with gray shaded line. Genes of interest within LD block shown as solid segments

Significant genome-wide associations were declared for both SNP-based and haplotype-based associations analyzed using hapQTL with a threshold of log_10_BF = 6.0. This threshold was confirmed by permuting case-control labels and examining the maximum null Bayes factors of the whole genome. The alternative and null distributions of Bayes factors showed unimodal distributions (Fig. 2A and B). The maximum log_10_BF values for the single-SNP test and the haplotype test were 5.286 and 1.647, respectively, implying that the set threshold was very stringent. All genome-wide significant associations for SNP-based and haplotype-based GWAS were demonstrated in Manhattan plots (Fig. 2A and B). Some genes in the MHC region were significantly detected by both single-SNP and haplotype tests, mainly including SNPs in *GABBR1*, *HLA-H*, *HLA-DPA1*, and *HLA-DPB1* on chromosome 6 (Table 1). The single SNP test identified SNPs in *BRAP* (log_10_BF = 8.726 and –log_10_p = 10.315) and *ALDH2* (log_10_BF = 7.406 and –log_10_p = 9.030) that were located on chromosome 12 associated with drug dependence, and the haplotype test found three SNPs in *TCN2* (log_10_BF = 6.077 and –log_10_p = 9.259), *SLC35E4* (log_10_BF = 7.607 and –log_10_p = 10.476) and *OSBP2* (log_10_BF = 6.768 and –log_10_p = 10.017) on chromosome 22 (Table 1). These results implied that these leading core SNPs with genome-wide significance may be associated with drug addiction.

**Table 1:** Genome-wide significant associations detected using hapQTL

SNP ID	Chr	Position	Gene	BF_2_	p_2_	BF_1_	p_1_
**Only detected by single SNP test**							
rs3782886	12	112,110,489	BRAP	4.131	5.768	8.726*	10.315
rs671	12	112,241,766	ALDH2	3.443	5.231	7.406*	9.030
**Only detected by haplotype test**							
rs9606757	22	31,016,645	TCN2	6.077*	9.259	−0.493	0.463
rs147307448	22	31,043,537	SLC35E4	7.607*	10.476	3.896	6.497
rs140377384	22	31,116,387	OSBP2	6.768*	10.017	0.344	2.049
**Detected by both single SNP and haplotype tests**							
rs29243	6	29,599,102	GABBR1	10.702*	12.379	11.548*	12.556
rs6937645	6	29,828,584	HLA-H	7.322*	8.160	6.005*	7.267
rs1431403	6	33,047,031	HLA-DPA1	7.023*	7.764	6.881*	9.814
rs3130188	6	33,057,176	HLA-DPB1	20.925*	23.685	15.135*	17.977

The leading core SNPs of each gene in this region were listed above. BF_2_ and p_2_ are log_10_BF and -log_10_p for haplotype test, and BF_1_ and p1 are log_10_BF and -log_10_p for single SNP test, respectively. The threshold for both BF_2_ and BF_1_ to declare genome-wide significant associations is 6, and * denotes genome-wide significant. The coordinates are from NCBI Build 37

For the screened candidate SNPs, we ultimately obtained 17 genes, many of which had multiple SNPs, especially *HLA-DPB1* [22], and *HLA-H* [19]. Various functional roles have been reported for these genes, including an association between the *HLA-DPB1* allele and the hepatitis B virus booster vaccination, while *HLA-H* is a membrane-bound ligand of Denisovan origin that protects against lysis by activated immune effector cells [23]. Functional analysis of these involved genes showed that they were enriched in several biological pathways via *HLA-H*, *HLA-DPB1*, and *HLA-DPA1*, mainly including the integral component of the lumenal side of the endoplasmic reticulum membrane, antigen processing and presentation, endoplasmic reticulum to Golgi transport vesicle membrane, and the interferon-gamma-mediated signaling pathway (Fig. 2C). These processes implicated the roles of involved genes, especially for their possible contributions via MHC molecules which have been found to play a critical role in various psychiatric illnesses [24]. Seven genes (41.18%) were specifically identified as members in multiple KEGG pathways, especially *ALDH2* [12], *HLA-DPA1* [9], and *HLA-DPB1* [9], suggesting that these genes have important roles in many biological processes (Fig. 2D).

### Screening and identification of causal haplotypes on chromosome 22

**Fig. 3:**
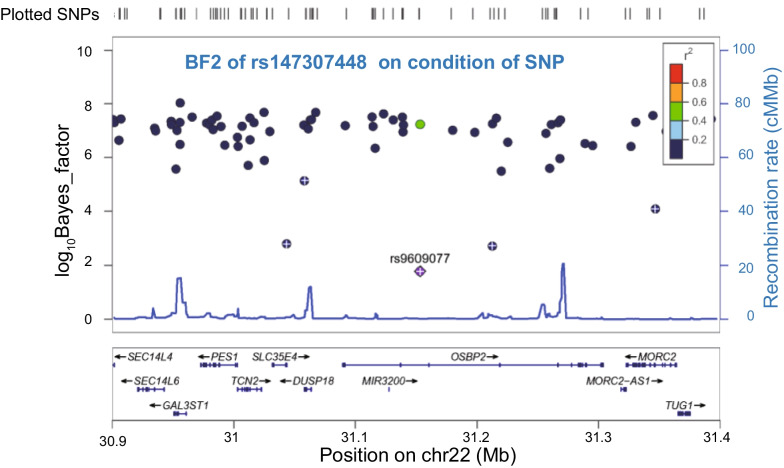
Association between local haplotype of rs147307448 and drug dependence on condition of each SNP. If the association of haplotype on condition of the SNP was still significant, this SNP was relatively independent of the haplotype of interest. The five SNPs denoted by white “+” may be vital to the haplotype, with lowest log_10_BF

**Fig. 4:**
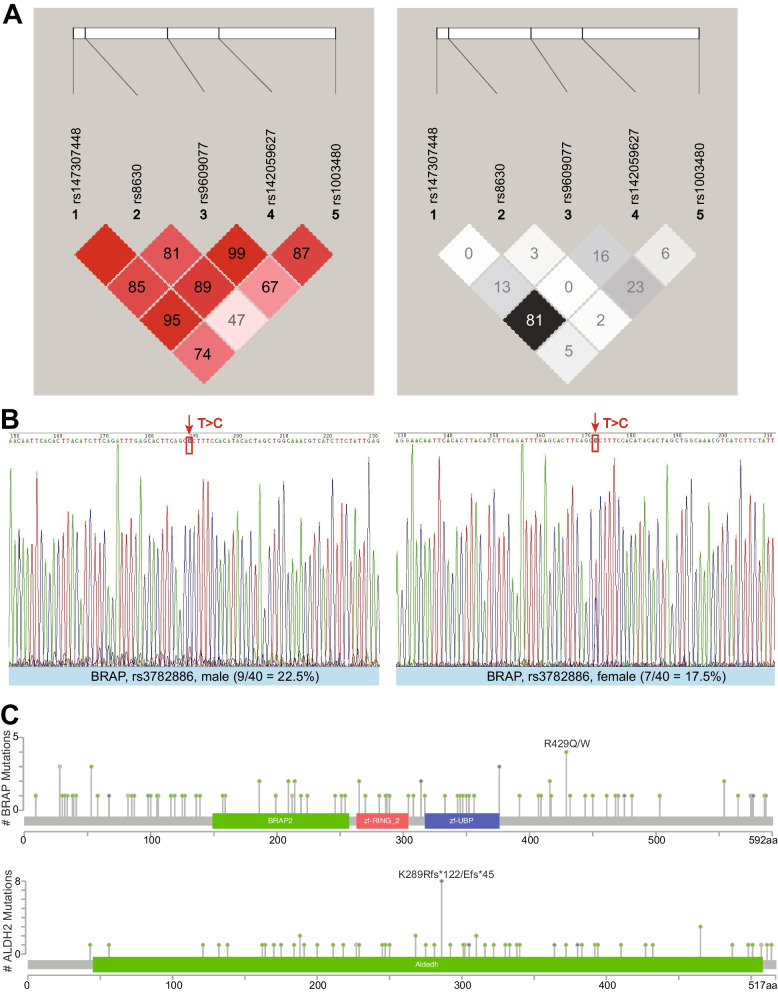
LD heatmaps and validation of candidate SNPs. **A** LD heatmaps of five tagged SNPs. Colors and values of LD heatmap in left panel correspond to D′ between markers, while those in right panel correspond to R2 between markers. D′ and R2 are widely used to evaluate LD but are poorly understood. D′ = 0.8 indicates high disequilibrium, indicating that two SNPs are co-inherited roughly 80% of the time. Low R2 in this strong LD also takes account of allele frequency. Low R2 indicates that SNPs cannot substitute for each other. Differences between D′ and R2 values also indicate a region with allelic heterogeneity. **B** Experimental and sequencing validation of rs3782886 in males and females, respectively. **C** Mutation profiles of *BRAP* and *ALDH2* in cancer

Based on a previous study [18], LD between markers can be quantified by local haplotype sharing (LHS), and the LD block around a leading core SNP can be defined as the largest region with LHS values > 2.5 × the background LHS values. Herein, core SNPs in genes within the LD block were identified on chromosome 22, mainly involving *SLC35E4, TCN2, OSBP2, DUSP18*, and *MORC2*, which showed significant haplotype associations (Fig. 2E and Table 1). The LD block inferred by LHS was from 30.9–31.4 Mb, containing 71 common SNPs (minor allele frequency > 0.01). We incorporated the genotype in 012 of one of the 71 SNPs as covariates, and then performed haplotype associations on the condition of a single SNP (Fig. 3 and Table S1). If the Bayes factor for the condition of a single SNP was still significant, this SNP was deemed to be relatively independent of the haplotype of interest; however, if the BF on condition of a SNP decreased dramatically, this SNP may be a vital to the haplotype. We finally selected the top three, five, and 11 vital SNPs, with the lowest BFs from Table S1. By incorporating 012 genotypes of the three, five, 11, and 71 SNPs into covariates respectively, haplotype association analysis on condition of multiple SNPs indicated that the top five vital SNPs could tag the haplotype well (Table S2). These five tagged SNPs, rs147307448, rs8630, rs9609077, rs142059627 and rs1003480, were all intron variants. The LD heatmaps of 5 tag SNPs were demonstrated (Fig. 4A), which indicates allelic heterogeneity of the locus. We then used Shapeit [25] to phase diploid genotypes into haplotypes and confirmed all possible haplotypes for the five tag SNPs (Table S3). We calculated and compared the frequencies of each haplotype in case and control samples, and finally identified three causal haplotypes of this region (Table S4), including AGACG and GGATG with protective roles, and GAGTA with a role in the risk of drug addiction.

### Verification by plink set-based tests and VEGAS2 gene-based tests

The genome-wide significant associations screened by SNP and haplotype tests were further verified by PLINK set-based tests and VEGAS2 gene-based tests (Table S5). Both set-based and gene-based tests are particularly suitable for large-scale candidate gene studies because they use permutation. The signals on Chr12, Chr22, and Chr6 can be verified well. For the signal on chromosome 22, among all sets in the LD block, the set of five SNPs was significant (*p* = 3.0e-09), further indicating that the five SNPs could mark the causal haplotype.

### Genetic correlations with other psychiatric phenotypes

We validated our screened results by estimating the genetic correlations with other GWAS results. There was a significant genetic correlation between drug dependence and MDD (Table S6, *p* = 0.002), but no significant correlation with other GWAS results. This was mainly due to differences among ethnic groups; we used European or East Asian LD scores, but these did not match the ancestry backgrounds of all participant samples. Nevertheless, the significant genetic correlation between drug dependence and MDD still provided strong evidence for the reliability of our GWAS findings.

### Experimental validation of candidate genes

We further validated the SNPs in genes associated with drug addiction, *BRAP* rs3782886 and *ALDH2* rs671, using an internal experimental validation using our cohort data. Sixteen samples (20%) were analyzed for *BRAP* rs3782886 (Fig. 4B), which has been shown to contribute to a predisposition for an alcohol use disorder [26]. *ALDH2* rs671 (G > A) was not detected, but there was an insertion mutation (Fig. 4C) after the A > C SNP in two samples (2.5%). There was strong LD values between *BRAP* rs3782886 and *ALDH2* rs671 [27], suggesting that they may have similar characteristics.

## Discussion

Drug addiction is indirectly and directly responsible for millions of deaths each year globally [28], accounting for more deaths than cancer, according to the Global Burden of Disease study. As a chronic, relapsing disease, drug addiction mainly results from the prolonged effects of drugs on the brain, and its treatment and management require personalized strategies [29]. Knowledge of the critical mechanisms underlying drug addiction is critical to the development of new preventive and therapeutic strategies [30]. We thus aimed to clarify the potential molecular features affecting drug addiction using SNP-based and haplotype-based association analyses. The haplotype method has more power for regions with allelic heterogeneity, while the single-SNP method performs better for regions without allelic heterogeneity. Both methods thus show unique and complementary detection abilities.

Among the significant associations detected by both SNP-based and haplotype-based methods, the *GABBR1* gene showed significant biological implications, and was stably expressed in case of alcohol dependence (Fig. S1B). *GABBR1* is an important regulator in the GABAergic system that helps to modulate neurotransmitter release and regulate the activity of ion channels and adenyl cyclase. The GABAergic system plays an important role in the mechanism of drug dependence and *GABBR1* has been associated with MA dependence and relapse after rehabilitation [31]. Furthermore, the novel associations between several human leucocyte antigen (HLA) genes, including HLA-H, HLA-DPA1, and HLA-DPB1, and drug dependence were also detected by both methods. Of these, HLA-DPA1 and HLA-DPB1 showed stable expression patterns in alcohol addicts (Fig. S1B). HLA is the specific term for the human MHC, which plays a vital role in the immune response, and contributes to the neuroimmune system in the development of drug addiction [25, 32]. The above HLA genes are also involved in multiple biological processes. The current findings thus confirm an underlying role for innate immune system underlies in drug addiction [22].

SNP-based findings showed that *BRAP* rs3782886 and *ALDH2* rs671 were associated with drug dependence, and the former is experimentally validated in the drug dependent population. *BRAP* rs3782886 was associated with the risk of metabolic syndrome in a young adult Chinese population [33], the risk of alcohol dependence and scores on the alcohol use disorders identification test [34], and activation of endothelial repair activity in elderly Japanese individuals. BRCA1-associated protein (*BRAP*) is a regulatory protein that binds to several translocation signal proteins in the cytoplasm and contributes to several intracellular signaling pathways, including the mitogen-activated protein kinase signaling pathway during central nervous system development as a ubiquitin ligase [35, 36], and nuclear factor kappa B (NF-κB) [37] as a primary mediator of inflammatory cascades. Alcohol and other abused drugs can induce NF-κB activity and cytokine expression in the brain [34, 38], indicating that the innate immune response is a causal link between NF-κB activation and substance abuse [36, 39]. NF-κB can also induce the expression of a diverse set of gene targets involved in addictive processes, such as opioid receptors and neuropeptides [40]. Furthermore, rs671 in *ALDH2* is involved in the alcohol flush reaction, also known as the “Asian Flush”. The association between *ALDH2* and drug dependence has been reported in the Chinese Han population [41]. The rs671 A allele may cause low *ALDH2* enzyme activity and reduced metabolism, resulting in delayed clearance of drugs and increased plasma drug concentrations. *ALDH2* is an important factor in opioid-use disorder and is associated with neuropsychological performance after methadone maintenance therapy [42]. There is thus compelling evidence indicating a role for *ALDH2* rs671 in drug dependency.

*BRAP* participates in the transduction of emergency signals and the regulation of physiological functions under emergency conditions, reduces intracellular reactive oxygen species and the level of intracellular oxidative metabolism, maintains the mitochondrial membrane potential under cell stress, reduces the activation of caspase-9, and inhibits cell apoptosis. *ALDH2* belongs to the nuclear protein-coding family, which has esterase, dehydrogenase, and reductase activities, and functions by entering mitochondria. *ALDH2* can activate the expression of cleaved caspase-3 protein, increase the ratio of Bcl / Bax, and play an important role in apoptosis. Abnormal *ALDH2* and *BRAP* alter the apoptotic signaling pathway resulting in hypoxia and ischemia in brain, increased oxygen free radicals, and increased oxidative stress. Excessive oxidative stress weakens the scavenging effects of superoxide dismutase and catalase [43], and impairs the integrity of cell membranes. In drug-dependent patients, oxidative stress will combine with the inflammatory response, promote platelet aggregation in damaged tissue, and aggravate cerebral ischemia and hypoxia, creating a vicious cycle [44].

The haplotype test showed an association between chromosome 22 and drug dependence, with potentially significant biological implications. Both *TCN2* and *SLC35E4* are involved in the “Cobalamin (vitamin B12) transport and metabolism” super pathway (Fig. S1C). *TCN2* encodes a member of the vitamin B12-binding protein family, which binds cobalamin and mediates its metabolism, and an association between *TCN2* and vitamin B12 deficiency has been widely reported by GWAS studies [45]. Vitamin B12 is essential for brain health, and vitamin B12 deficiency may contribute to addiction; some MA addicts showed vitamin B12 deficiency, and serum B12 levels may be involved in the prognosis of MA addiction [46]. The current results imply that this region with allelic heterogeneity may be involved in drug dependence via vitamin B12. A Chinese population study also reported that some MA addicts showed vitamin B12 deficiency, and suggested that serum B12 levels may be involved in the prognosis of MA addiction [25]. In addition, a previous review showed that vitamin B12 deficiency was more common in women with, than in those without an opioid use disorder [32]. On this basis, *TCN2* and *SLC35E4* and their local haplotypes may be involved in drug dependence via vitamin B12.

Overall, the current SNP-based and haplotype-based analyses indicated that immune-related genes in the MHC, and *BRAP* rs3782886 and *ALDH2* rs671, may be associated with drug dependence. These findings will help to reveal crucial susceptibility genes underlying drug addiction. These related genes have critical roles in multiple biological processes, and may thus provide new insights into effective strategies for the prevention and treatment of addiction.

### Supplementary Information

**Supplementary Material 1.**

## Data Availability

The data could be available from corresponding author due to reasonable request.
