# Supplementary material for: SNP-based and haplotype-based genome-wide association on drug dependence in Han Chinese
Source: BMC Genomics. 2024 Mar 6;25:255. doi: 10.1186/s12864-024-10117-4 (PMC10919046; doi:10.1186/s12864-024-10117-4)
Supplement: Supplementary file 1 — Supplementary Material 1. [file 12864_2024_10117_MOESM1_ESM.docx]

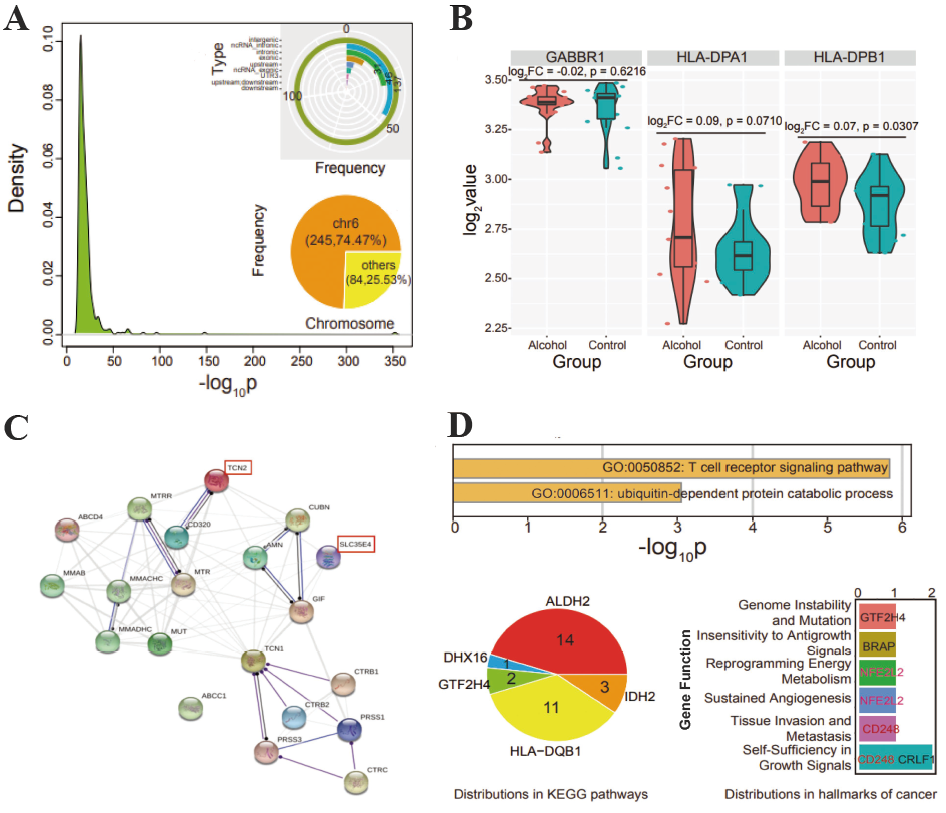


**Figure S1. Distribution and function analysis for screened genes**

1. Distribution of p-values for all screened significantly varied sites (p <= 9.96e-07). The pie chart shows the dominant distribution on chromosome 6 (245 varied sites are located on chromosome 6), and the detailed distribution types are also shown.
2. Expression distributions of several genes in alcohol and control samples, and p-values are estimated using t-test (alcohol group = 18, control group = 17).
3. STRING interaction network for cobalamin (vitamin B12) transport and metabolism SuperPath. *TCN2* and *SLC35E4* are both involved in the pathway, indicated by red rectangles.
4. The significantly enriched GO terms for genes detected by PLINK, and the potential contributions in KEGG pathways.

**Table S1. Haplotype association tests on condition of single SNP**

| **Condition on SNP** | | | **BF_2_ of rs9606757** | **BF_2_ of rs147307448** | **BF_2_ of rs140377384** |
| --- | --- | --- | --- | --- | --- |
| \ | | | 6.077 | 7.339 | 6.768 |
| rs9608955 | chr22 | 30900829 | 6.224 | 7.411 | 6.797 |
| rs9608956 | chr22 | 30901592 | 6.084 | 7.300 | 6.687 |
| rs933339 | chr22 | 30905491 | 5.434 | 6.644 | 5.947 |
| rs2023967 | chr22 | 30907168 | 6.244 | 7.438 | 6.807 |
| rs118116676 | chr22 | 30934885 | 5.864 | 7.096 | 6.507 |
| rs5753194 | chr22 | 30935682 | 5.744 | 6.996 | 6.607 |
| rs182798258 | chr22 | 30948498 | 6.084 | 7.349 | 6.777 |
| rs6518702 | chr22 | 30948752 | 5.724 | 7.226 | 6.377 |
| rs2267159 | chr22 | 30952396 | 4.644 | 5.574 | 5.127 |
| rs2267161 | chr22 | 30953295 | 5.374 | 7.012 | 6.527 |
| rs118120731 | chr22 | 30955669 | 6.034 | 7.308 | 6.607 |
| rs5749126 | chr22 | 30955722 | 5.254 | 6.489 | 5.797 |
| rs12628608 | chr22 | 30955907 | 6.814 | 8.034 | 7.097 |
| rs42938 | chr22 | 30965757 | 6.404 | 7.509 | 6.777 |
| rs142214789 | chr22 | 30977576 | 5.974 | 7.282 | 6.667 |
| rs191006503 | chr22 | 30979921 | 6.024 | 7.222 | 6.607 |
| rs111338112 | chr22 | 30981984 | 6.224 | 7.397 | 6.707 |
| rs377738551 | chr22 | 30983044 | 5.734 | 7.045 | 6.607 |
| rs4820018 | chr22 | 30986172 | 6.434 | 7.544 | 6.837 |
| rs11912615 | chr22 | 30989492 | 5.924 | 7.157 | 6.497 |
| rs4820876 | chr22 | 30992672 | 5.274 | 6.463 | 5.717 |
| rs5753231 | chr22 | 31003069 | 5.624 | 6.759 | 6.247 |
| rs16988828 | chr22 | 31003561 | 5.604 | 6.419 | 5.617 |
| rs9606756 | chr22 | 31006860 | 6.024 | 7.166 | 6.447 |
| rs5749135 | chr22 | 31011906 | 5.014 | 5.707 | 4.727 |
| rs2283873 | chr22 | 31013296 | 6.424 | 7.476 | 6.717 |
| rs117458738 | chr22 | 31013374 | 5.754 | 6.662 | 6.167 |
| rs9606757 | chr22 | 31016645 | 6.114 | 7.298 | 6.657 |
| rs9306264 | chr22 | 31024934 | 6.644 | 7.685 | 6.707 |
| rs731991 | chr22 | 31025189 | 5.124 | 5.893 | 4.527 |
| rs184886704 | chr22 | 31029674 | 5.774 | 6.970 | 6.587 |
| rs147307448 | chr22 | 31043537 | 2.174 | 2.805 | 2.677 |
| rs181701741 | chr22 | 31057697 | 6.024 | 7.208 | 6.577 |
| rs8630 | chr22 | 31058115 | 4.364 | 5.138 | 3.917 |
| rs67153574 | chr22 | 31061283 | 5.684 | 7.071 | 6.317 |
| rs117728004 | chr22 | 31063007 | 6.104 | 7.403 | 7.067 |
| rs3827379 | chr22 | 31063939 | 6.114 | 7.415 | 7.027 |
| rs9606764 | chr22 | 31067427 | 6.284 | 7.682 | 7.757 |
| rs117344502 | chr22 | 31091747 | 5.904 | 7.189 | 6.837 |
| rs11089471 | chr22 | 31113828 | 6.214 | 7.515 | 7.307 |
| rs58884052 | chr22 | 31114622 | 5.494 | 7.160 | 6.437 |
| rs140377384 | chr22 | 31116387 | 5.644 | 6.349 | 6.267 |
| rs136369 | chr22 | 31123292 | 6.304 | 7.628 | 7.357 |
| rs139174051 | chr22 | 31131227 | 6.104 | 7.401 | 7.317 |
| rs141280512 | chr22 | 31138802 | 6.124 | 7.511 | 7.287 |
| rs926340 | chr22 | 31139318 | 5.484 | 6.955 | 6.397 |
| rs5753303 | chr22 | 31139653 | 5.824 | 7.215 | 6.807 |
| rs9609077 | chr22 | 31153162 | 1.044 | 1.779 | 1.527 |
| rs9609078 | chr22 | 31153276 | 5.974 | 7.238 | 6.737 |
| rs117498931 | chr22 | 31179867 | 5.674 | 7.018 | 6.697 |
| rs136262 | chr22 | 31198037 | 5.604 | 6.938 | 7.307 |
| rs142059627 | chr22 | 31212513 | 2.184 | 2.719 | 2.297 |
| rs738374 | chr22 | 31212641 | 5.874 | 7.255 | 6.887 |
| rs361844 | chr22 | 31215815 | 6.334 | 7.469 | 6.937 |
| rs136223 | chr22 | 31219869 | 4.534 | 5.496 | 5.357 |
| rs140951642 | chr22 | 31225189 | 5.364 | 6.571 | 5.817 |
| rs4577391 | chr22 | 31256584 | 5.614 | 6.899 | 6.437 |
| rs5753355 | chr22 | 31259658 | 4.554 | 5.600 | 5.067 |
| rs142720771 | chr22 | 31261083 | 6.084 | 7.231 | 6.407 |
| rs2301816 | chr22 | 31266546 | 6.044 | 7.297 | 6.527 |
| rs2285917 | chr22 | 31268091 | 4.884 | 5.964 | 5.537 |
| rs112446420 | chr22 | 31268556 | 6.164 | 7.404 | 6.777 |
| rs11089481 | chr22 | 31288769 | 5.574 | 6.524 | 5.817 |
| rs5749191 | chr22 | 31295082 | 5.524 | 6.446 | 5.687 |
| rs143900220 | chr22 | 31326576 | 5.284 | 6.414 | 5.827 |
| rs118054267 | chr22 | 31330594 | 6.024 | 7.314 | 6.847 |
| rs117178020 | chr22 | 31344684 | 6.284 | 7.568 | 7.147 |
| rs1003480 | chr22 | 31346752 | 3.344 | 4.087 | 3.377 |
| rs2285919 | chr22 | 31355635 | 5.714 | 6.973 | 6.437 |
| rs56686418 | chr22 | 31388971 | 5.814 | 7.072 | 6.667 |
| rs9621153 | chr22 | 31392606 | 6.204 | 7.434 | 6.887 |

BF_2_ is log_10_(Bayes factor) of haplotype association. “\” stands for not on condition of any SNP. Top 3 SNPs are red. Top 5 SNPs are yellow.

**Table S2. Haplotype association tests on condition of multiple SNPs**

| **Condition** | **BF_2_ of rs9606757** | **BF_2_ of rs147307448** | **BF_2_ of rs140377384** |
| --- | --- | --- | --- |
| \ | 6.077* | 7.339* | 6.768* |
| 3 SNPs | 0.051 | 0.384 | 0.516 |
| 5 SNPs | -0.606 | -0.389 | -0.855 |
| 11 SNPs | -0.389 | 0.255 | 0.047 |
| 71 SNPs | -0.410 | 0.067 | 0.197 |

For the region with allelic heterogeneity on chromosome 22 we performed haplotype association tests on condition of multiple SNPs. The line with "\" is haplotype association not conditioned on any SNP, while the other lines are haplotype association conditioned on multiple SNPs. BF_2_ is log_10_BF for haplotype test, the threshold of BF_2_ to declare genome-wide significant associations is 6. BF_2_ on condition of 5 SNPs were the lowest. Intuitively, the 5 SNPs can tag this local haplotype well.

**Table S3. Frequencies of haplotypes on 5 tag SNPs in case and control samples**

|  | **Chromosome** | **Position** | **Gene** |
| --- | --- | --- | --- |
| rs147307448 | chr22 | 31043537 | SLC35E4 |
| rs8630 | chr22 | 31058115 | SLC35E4/DUSP18 |
| rs9609077 | chr22 | 31153162 | OSBP2 |
| rs142059627 | chr22 | 31212513 | OSBP2 |
| rs1003480 | chr22 | 31346752 | MORC2 |

**Table S4.** **Frequencies of haplotypes on all SNPs in case and control samples**

| Haplotype | Case (N=962🞨2) | | Control (N=8183🞨2) | | p-value | Type |
| --- | --- | --- | --- | --- | --- | --- |
|  | n | Freq | n | Freq |  |  |
| AGACG | 29 | 0.0151 | 515 | 0.0315 | 1.9e-05 | Protective |
| GAGTA | 372 | 0.1933 | 2566 | 0.1568 | 5.3e-05 | Risky |
| GGATG | 151 | 0.0785 | 1636 | 0.1000 | 0.002 | Protective |
| AGACA | 1 | 0.0005 | 57 | 0.0035 | 0.028 |  |
| GGGTA | 1002 | 0.5208 | 8323 | 0.5086 | 0.311 |  |
| GGACG | 0 | 0.0000 | 13 | 0.0008 | 0.385 |  |
| AGGTG | 0 | 0.0000 | 9 | 0.0005 | 0.611 |  |
| AGATG | 0 | 0.0000 | 11 | 0.0007 | 0.619 |  |
| GGGTG | 240 | 0.1247 | 2085 | 0.1274 | 0.772 |  |
| GAGTG | 46 | 0.0239 | 407 | 0.0249 | 0.877 |  |
| AGGTA | 7 | 0.0036 | 68 | 0.0042 | 1.000 |  |
| GGATA | 65 | 0.0338 | 558 | 0.0341 | 1.000 |  |
| GAATA | 7 | 0.0036 | 62 | 0.0038 | 1.000 |  |
| GAATG | 4 | 0.0021 | 40 | 0.0024 | 1.000 |  |
| GAACG | 0 | 0.0000 | 7 | 0.0004 | 1.000 |  |
| GGACA | 0 | 0.0000 | 2 | 0.0001 | 1.000 |  |
| AGATA | 0 | 0.0000 | 4 | 0.0002 | 1.000 |  |
| GGGCG | 0 | 0.0000 | 3 | 0.0002 | 1.000 |  |

The 5 tag SNPs are rs147307448 (chr22:31043537, SLC35E4); rs8630 (chr22:31058115, SLC35E4/DUSP18); rs9609077 (chr22:31153162, OSBP2); rs142059627 (chr22:31212513, OSBP2); rs1003480 (chr22:31346752, MORC2). The frequencies of each haplotype on 5 tag SNPs in case and control samples are calculated and compared. Three causal haplotypes are finally identified, namely AGACG，GAGTA and GGATG, respectively.

**Table S5. Verification of signals on Chr12, Chr22, and Chr6 using Plink set-based tests and VEGAS2 gene-based tests**

| **Signal** | **SNP set** | **Plink set-based test** | | **VEGAS2** | | |
| --- | --- | --- | --- | --- | --- | --- |
|  |  | **nSNPs** | **Set p-value** | | **nSNPs** | **Gene p-value** |
| Chr12 | BRAP | 3 | 2.6e-07 | | 3 | <1.0e-06 |
|  | ALDH2 | 10 | 2.3e-05 | | 10 | 2.0e-05 |
| Chr22 | 5 tag SNPs of the LD block | 5 | 3.0e-09 | | \ | \ |
|  | SNPs within the LD block | 71 | 7.7e-08 | | \ | \ |
|  | OSBP2 | 26 | 1.4e-05 | | 26 | 4.0e-05 |
|  | SLC35E4 | 6 | 1.9e-05 | | \ | \ |
|  | MORC2 | 5 | 5.5e-05 | | 5 | 1.5e-05 |
|  | TCN2 | 7 | 0.001 | | 4 | 1.7e-04 |
|  | DUSP18 | 4 | 0.004 | | 3 | 0.004 |
| Chr6 | HLA-H | 339 | 2e-08 | | \ | \ |
|  | GABBR1 | 78 | 4e-08 | | \ | \ |
|  | HLA-DPA1 | 60 | 0.009 | | 18 | 0.239 |
|  | HLA-DPB1 | 100 | 0.012 | | 2 | 0.146 |

A SNP set is a group of SNPs within a gene or a certain region, nSNP is the number of SNPs in the set. The numbers of permutations are 1.0e+9 and 1.0e+6 for set-based and gene-based tests respectively. The recommended significant threshold of p-value for set-based and gene-based tests is 10e-06. Therefore, *BRAP*, 5 tag SNPs of the LD block, *HLA-H*, and *HLA-DPB1* are genome-wide significant.

**Table S6. Genetic correlations between our GWAS findings and other GWAS results**

|  | **MDD** | **BIP** | **Cannabis** | **Alcohol** |
| --- | --- | --- | --- | --- |
| Genetic correlation | 0.29 | 0.04 | 0.11 | 0.28 |
| p value | 0.002* | 0.194 | 0.604 | 0.129 |

The genetic correlation of drug dependence and major depressive disorder (MDD) is significantly positive.
